# Supplementary material for: miR-7 controls glutamatergic transmission and neuronal connectivity in a Cdr1as-dependent manner
Source: EMBO Rep. 2024 Jun 3;25(7):3008–39. doi: 10.1038/s44319-024-00168-9 (PMC11239925; doi:10.1038/s44319-024-00168-9)
Supplement: Supplementary file 1 — Appendix [file 44319_2024_168_MOESM1_ESM.pdf]

Table of content

Figure	Page number
Appendix Figure S1	p6, p7
Appendix Figure S2	p7
Appendix Figure S3	p11, pp12
Appendix Figure S4	p14, p15
Appendix Figure S5	p17
Appendix Figure S6	p23
Extended Discussion	p22
Appendix Figure S7	p23

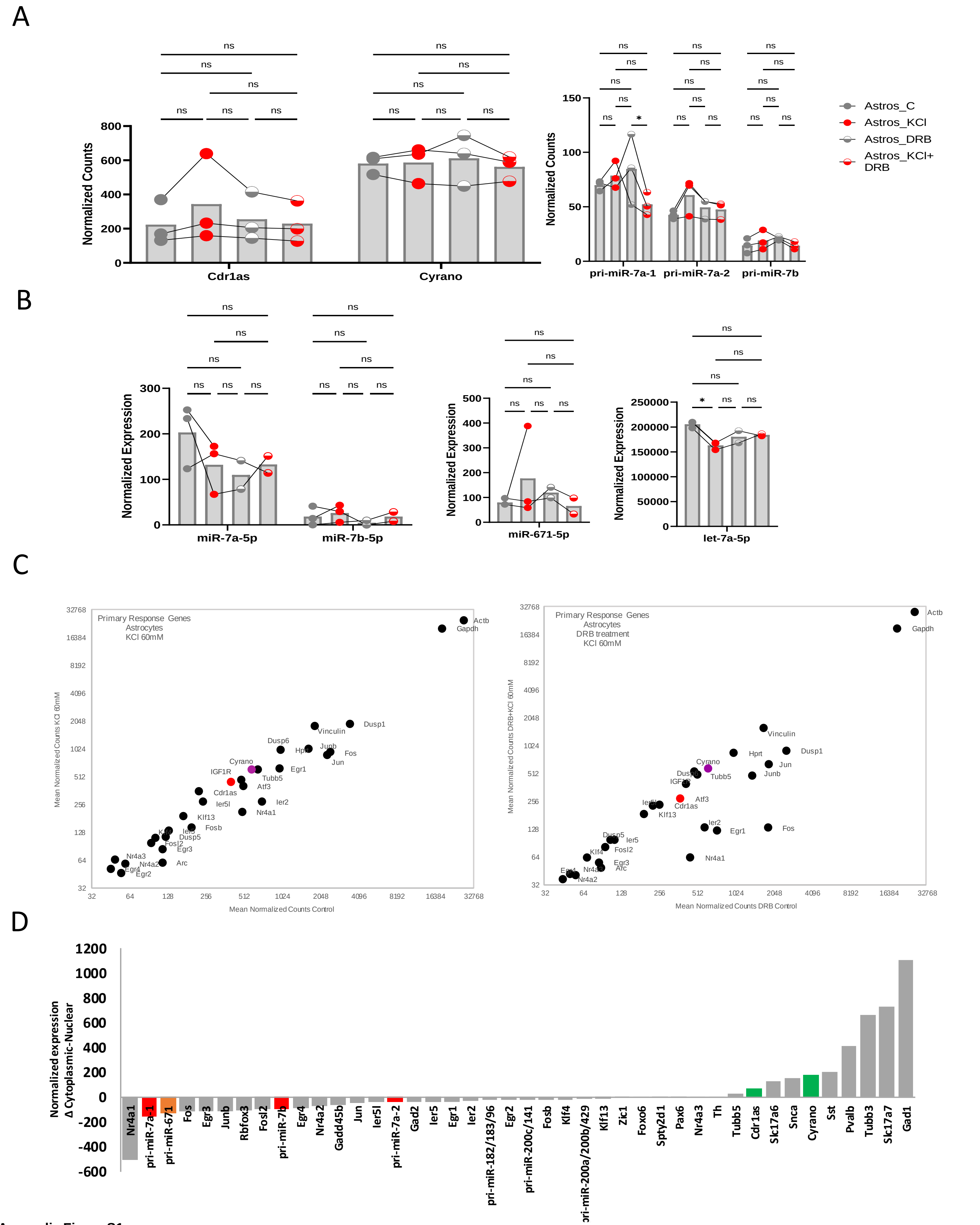

Appendix Figure S1.

(A) Quantification of Cdr1as, Cyrano, and primary miRNAs (Nanostring nCounter methods), in primary astrocytes exposed to neuronal culture (feeder layer) before and after sustained depolarization plus pre-incubation with transcription inhibitor (DRB). RNA counts are normalized to housekeeping genes (Actb, Tubb5, and Vinculin). Bar plot represents the mean of 3 biological replicates (3 independent primary cultures from 3 animals). P-value: two-way ANOVA.

(B) Expression levels of mature miR-7 isoforms (left), miR-671 (middle), and let-7a (right) quantified by small RNA-seq for 3 independent primary cultures, in primary astrocytes exposed to neuronal culture (feeder layer) before and after sustained depolarization plus pre-incubation with transcription inhibitor (DRB). Bar plot represents the mean. P-value: two-way ANOVA. Error: SD.

(C) RNA quantification of IEGs in primary astrocytes exposed to neuronal culture (feeder layer) before and after sustained depolarization (left) plus pre-incubation with transcription inhibitor (DRB) (right)(Nanostring nCounter, methods). Cdr1as and Cyrano shown in red and purple, respectively. RNA counts are normalized to housekeeping genes (*Actb, Tubb5 and Vinculin*). Each dot represents the mean of 3 biological replicates (3 independent primary cultures from 3 animals).

(D) RNA quantification of genes of interest (Nanostring nCounter, methods) in nuclear versus cytoplasmatic fractions of WT neurons DIV21. Each bar represents the mean difference of 3 independent biological replicates. Cdr1as and Cyrano: green. Primary miRNAs: red and orange. RNA counts are normalized to housekeeping genes (*Actb, Tubb5 and Vinculin*).

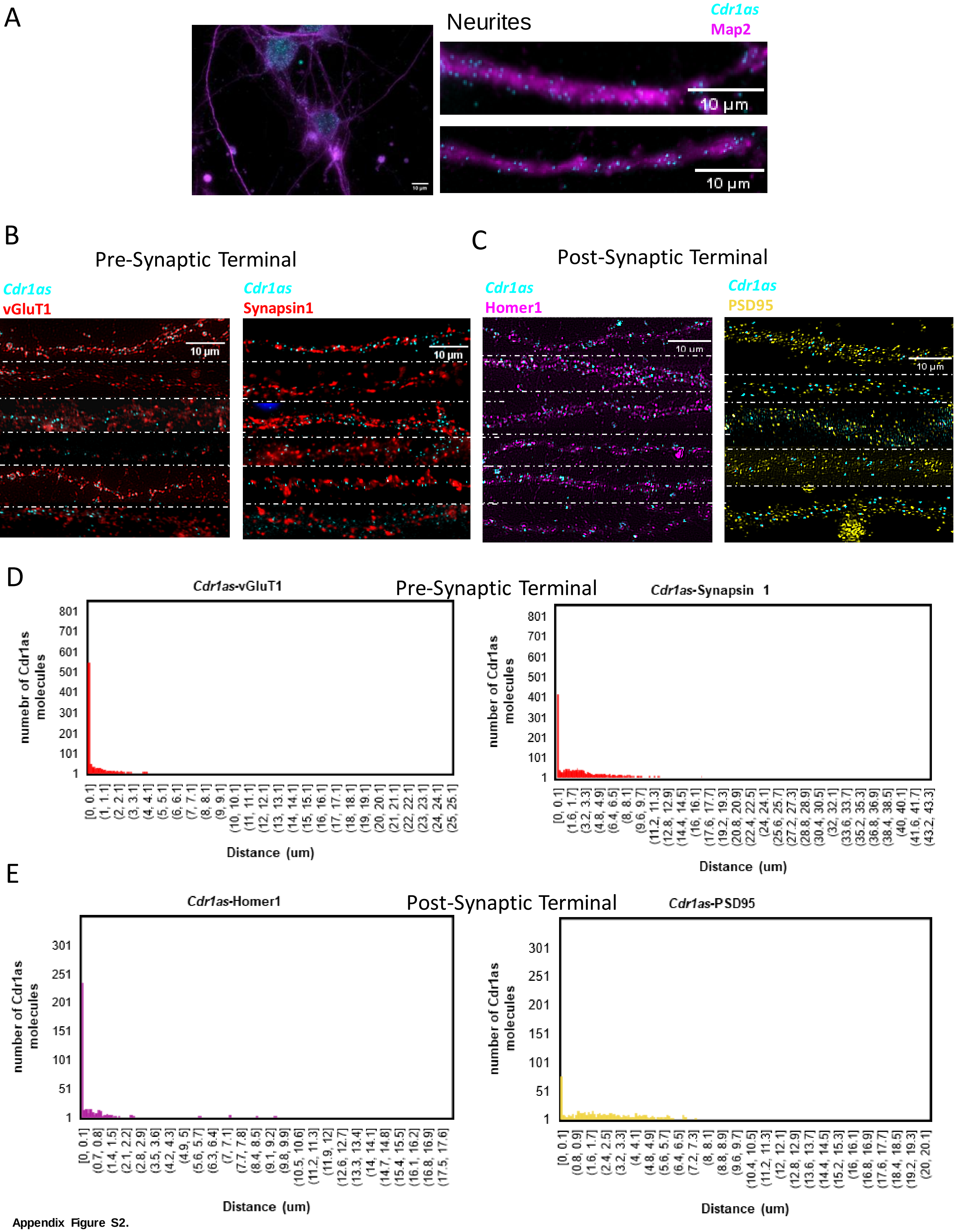

Appendix Figure S2.

(A) Left: Single molecule RNA FISH combined with immunofluorescence (Stellaris+IF, Methods) of *Cdr1as* RNA (cyan) and Map2 protein (magenta) performed in WT neurons DIV21. Right: Single neurite zoom in.

(B) Single neurites zoom in. Showed by single molecule RNA FISH combined with immunofluorescence (Stellaris+IF, Methods) is the co-localization of *Cdr1as* RNA (cyan) with different pre-synaptic terminal proteins: vGLuT1 (red, left), Synapsin1 (red, right).

(C) Representative single neurites zoom in. Showed by single molecule RNA FISH combined with immunofluorescence (Stellaris+IF, Methods) is the co-localization of *Cdr1as* RNA (cyan) with different synaptic post-synaptic terminal proteins: Homer1 (magenta), PDS95 (yellow).

(D) Quantification of colocalization of *Cdr1as* molecules with a specific pre-synaptic protein (Methods). Measure of the distance from each Cdr1as molecule's center to its nearest protein of interest. Histogram: distances derived from all images are plotted. Cdr1as-vGLuT1 (left): 14 images. Cdr1as-Synapsin1 (right): 37 images.

(E) Quantification of colocalization of *Cdr1as* molecules with a specific post-synaptic protein (Methods). Measure of the distance from each Cdr1as molecule's center to its nearest protein of interest. Histogram: distances derived from all images are plotted. Cdr1as-Homer1 (left): 11 images. Cdr1as-PSD95 (right): 16 images.

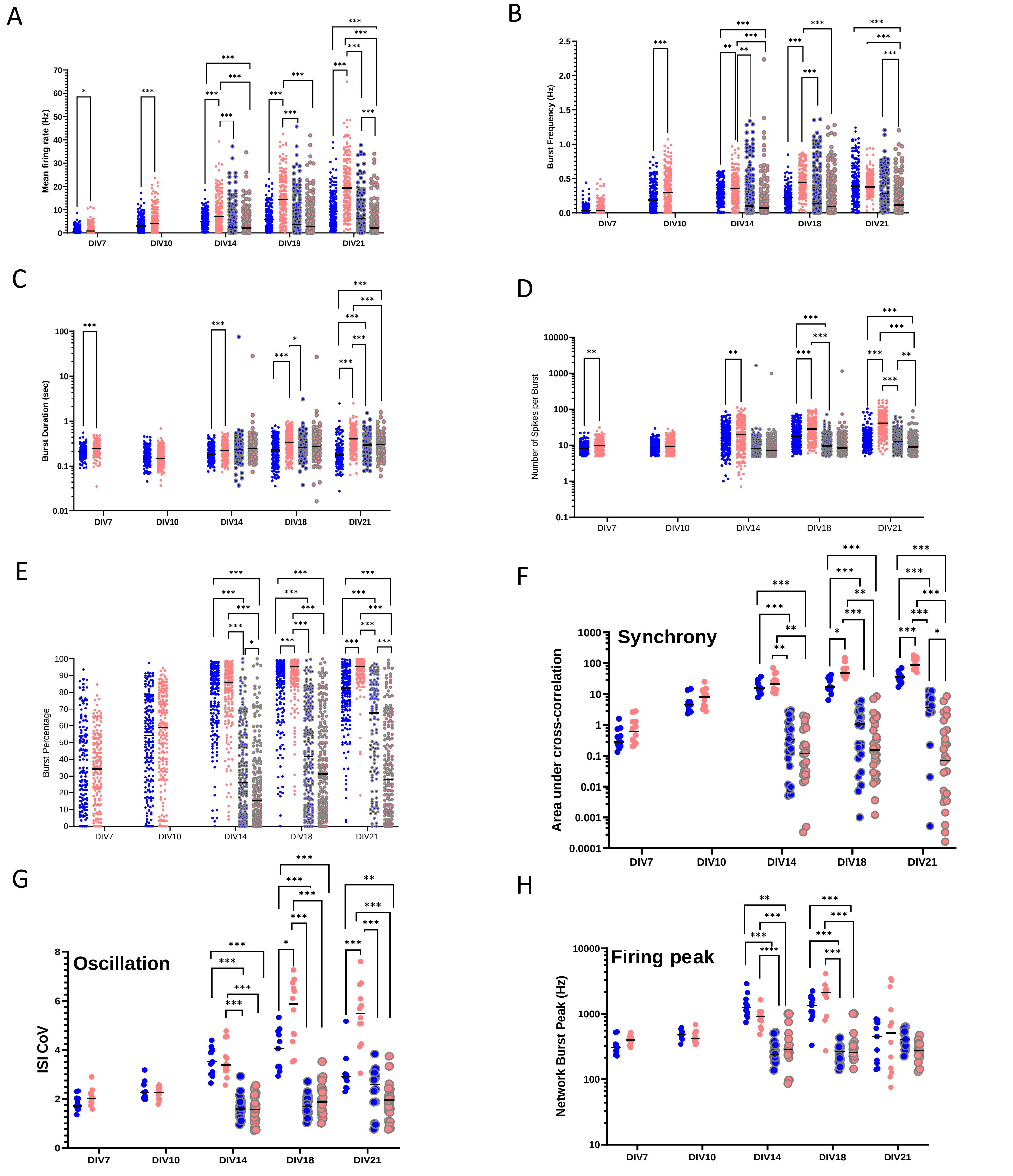

Appendix Figure S3.

(A) Mean Firing Rate: Total number of spikes per single-electrode divided by the duration of the analysis (600s), in Hz. Each dot represents a single electrode recording from 4 independent primary cultures (WT = 187; WT + miR-7 overexpression = 194; Cdr1as-KO = 189; Cdr1as-KO + miR-7 overexpression = 200 electrodes). Horizontal line: median. P value: two-way ANOVA; Multiple comparison correction test: Bonferroni, all other comparisons were not statistically significant (not shown).

(B) Burst Frequency: Total number of single-electrode bursts divided by the duration of the analysis, in Hz. Panel plotted as in (A).

(C) Burst Duration: Average time (sec) from the first spike to last spike in a single-electrode burst. Panel plotted as in (A).

(D) Number of Spikes per Burst: Average number of spikes in a single-electrode burst. Panel plotted as in (A).

(E) Burst Percentage: number of spikes in single-electrode bursts divided by the total number of spikes, multiplied by 100. Panel plotted as in (A).

(F) Asynchrony: The ability of neurons to generate APs simultaneously was calculated as area under the well-wide pooled inter-electrode cross-correlation. Higher areas indicate lower synchrony (Halliday et al., 2006, Methods). Each dot represents a network recording from 4 independent primary cultures (WT = 11; WT + miR-7 overexpression = 26; Cdr1as-KO = 12; Cdr1as-KO + miR-7 overexpression = 28 electrodes); All metrics apply to network bursts across single wells within 20 ms. P value: two-way ANOVA. Multiple comparison correction test: Bonferroni, all other comparisons were not statistically significant (not shown).

(G) Oscillation: Average across network bursts of the inter spike interval coefficient of variation (ISI CoV) (standard deviation/mean of the inter-spike interval) within network bursts. Oscillation is a measure of how the spikes from all of the neurons are organized in time. WT = 11-26; Cdr1as-KO: 12-28 independent network recordings. Panel plotted as in (F).

(H) Burst Peak: Maximum number of spikes per second in the average network burst. The peak of the average network burst histogram divided by the histogram bin size to yield spikes per sec (Hz). WT = 11-26; Cdr1as-KO: 12-28 independent network recordings. Panel plotted as in (F).

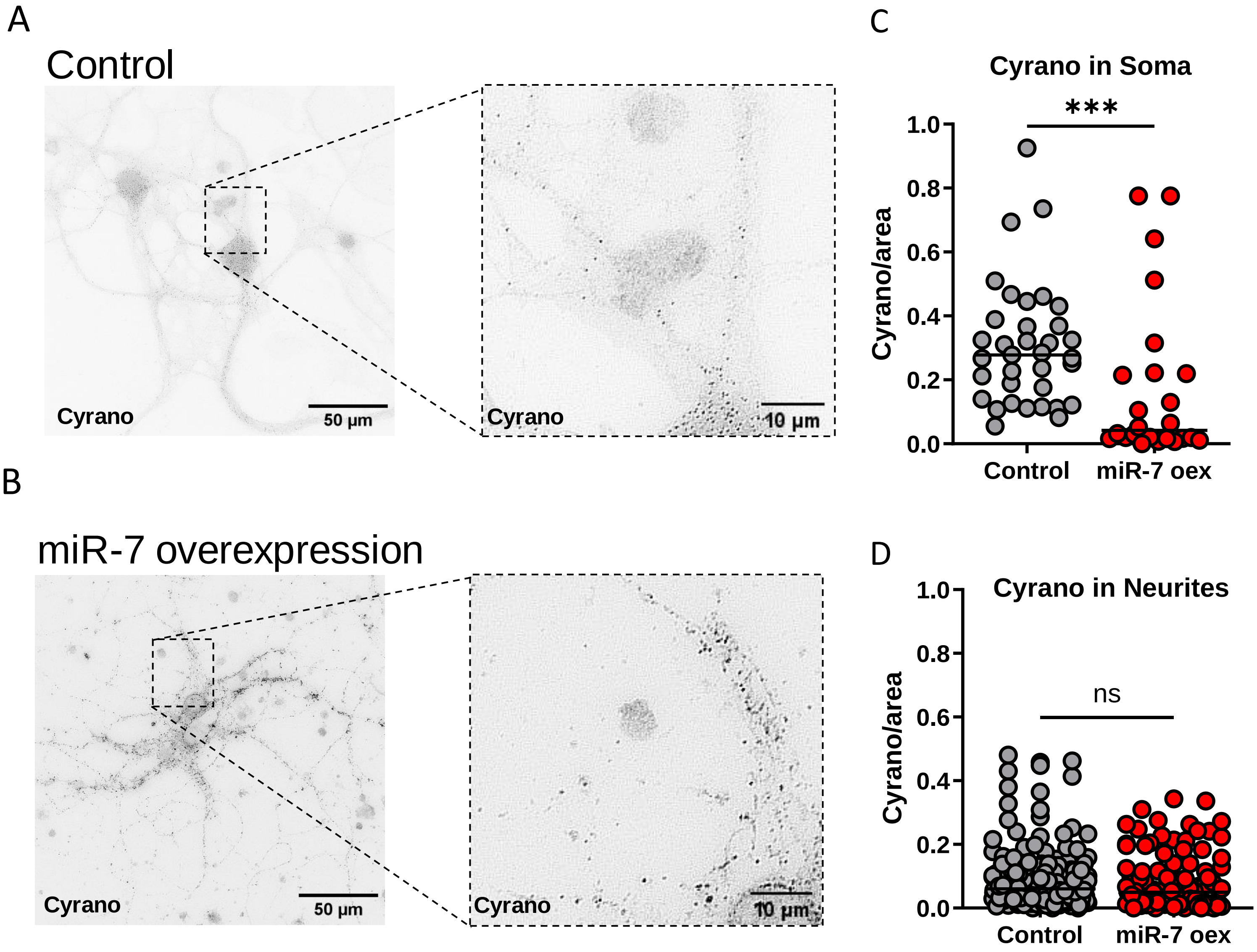

Appendix Figure S4.

(A) Single molecule RNA FISH (Stellaris, Methods) of Cyrano (black dots) performed in WT neurons DIV21 from control condition. Left: single neuron at 60X. Right: zoom in of soma-neurite compartment.

(B) Single molecule RNA FISH (Stellaris, Methods) of Cyrano (black dots) performed in WT neurons DIV21 infected with control or miR-7a overexpression construct. Left: neurons imaged at 60X. Right: zoom in of soma-neurite compartment.

(C) smRNA FISH quantification of Cyrano molecules (following Raj et al., 2008, Methods). Each dot represents the mean number of Cyrano molecules in an independent soma normalized by area (100 px = 21,5 um). Control neurons: 25 somas (2 independent cultures from 2 animals); miR-7 overexpression neurons: 35 soma (2 independent cultures from 2 animals). Horizontal line: Median. P value: U Mann–Whitney test (nonparametric, unpaired).

(D) smRNA FISH quantification of Cyrano molecules (following Raj et al., 2008, Methods). Each dot represents the mean number of Cyrano molecules in an independent cell neurite normalized by area (100 px = 21,5 um). Control neurons: 110 neurites (2 independent cultures from 2 animals); miR-7 overexpression neurons: 230 neurites (2 independent cultures from 2 animals). Horizontal line: Median. P value: U Mann–Whitney test (nonparametric, unpaired).

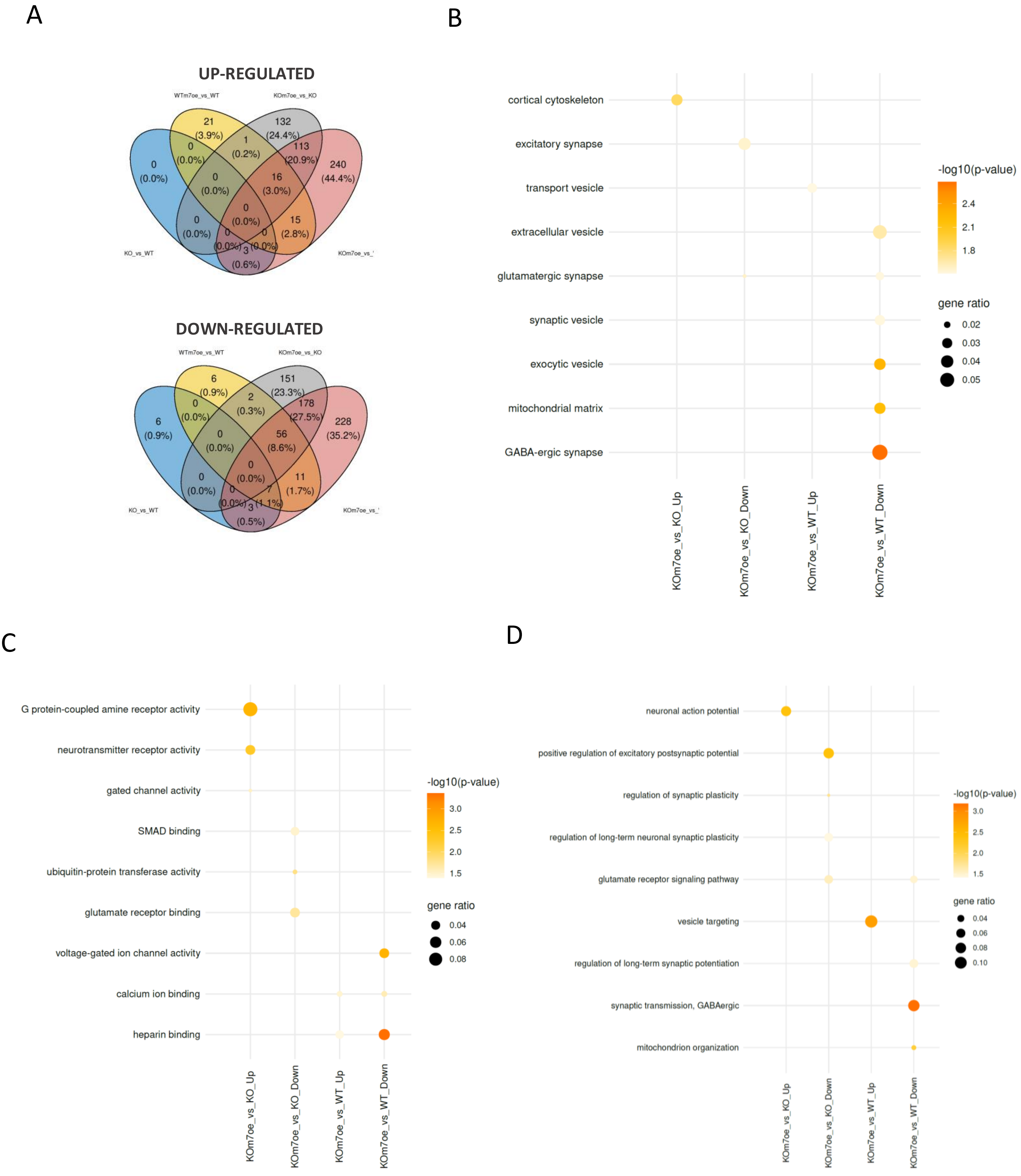

Appendix Figure S5.
(A) Number of specific and commonly up-regulated and down-regulated genes from mRNA expression changes for each comparison tested (Cdr1as-KO vs WT, WTmiR-7oex. vs WT, Cdr1as-KO miR-7oex.vs Cdr1as-KO). Venn diagram, mean change of 4 independent biological replicates per condition.
(B) Enrichment profile from gene ontology (GO) analysis of cellular compartments (CC). Gene ontology enrichment analysis done using topGOtable function in the pcaExplorer (Marini et al., 2019, Methods). The dot size shows gene ratio and the color denotes the FDR-corrected p-value. Genes with average log2 fold change of 0.5 and adjusted p-value less than 0.05 were considered significant and all expressed genes were used as background.
(C) Enrichment profile from gene ontology (GO) analysis of Molecular Functions (ML). Enrichment profile from GO analysis plotted as in (B)
(D) Enrichment profile from gene ontology (GO) analysis of Biological Process (BP). Enrichment profile from GO analysis plotted as in (B)

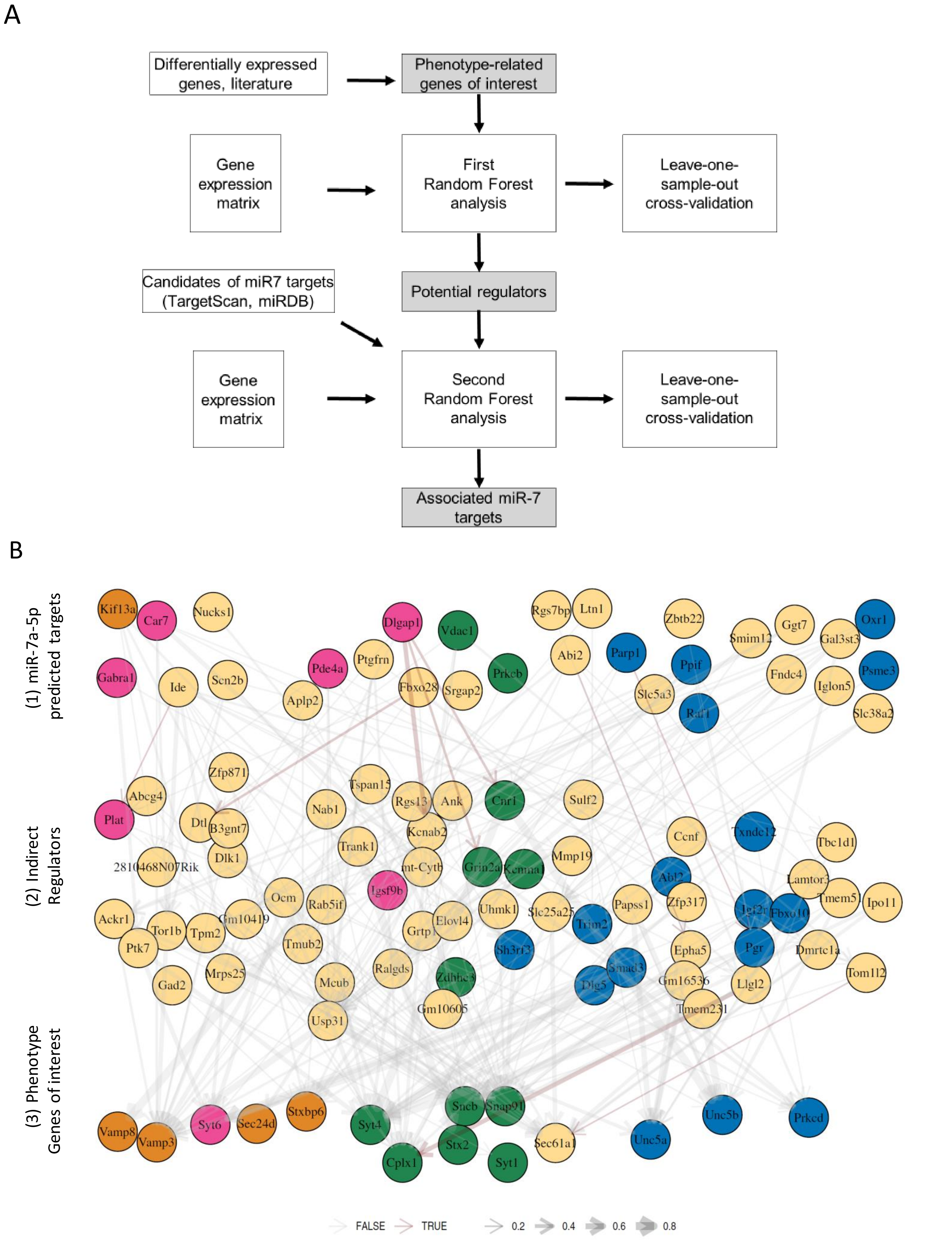

Appendix Figure S6.

(A) Schematic representation of Random Forest modelling methodology.

(B) Consensus gene regulatory network (GRN) of miR-7 and Cdr1as in neurons. The first layer are miR-7 target genes, the middle layer are intermediate regulator genes, and the bottom layer are phenotype-related genes from literature. The node color indicates gene ontology terms associated with synaptic (pink), vesicle (orange), apoptosis (blue), and common (green), respectively. The edge width corresponds to the relative fraction of cross-validation models having this edge (Methods). Colored edges indicate links found in StringDB (Methods).

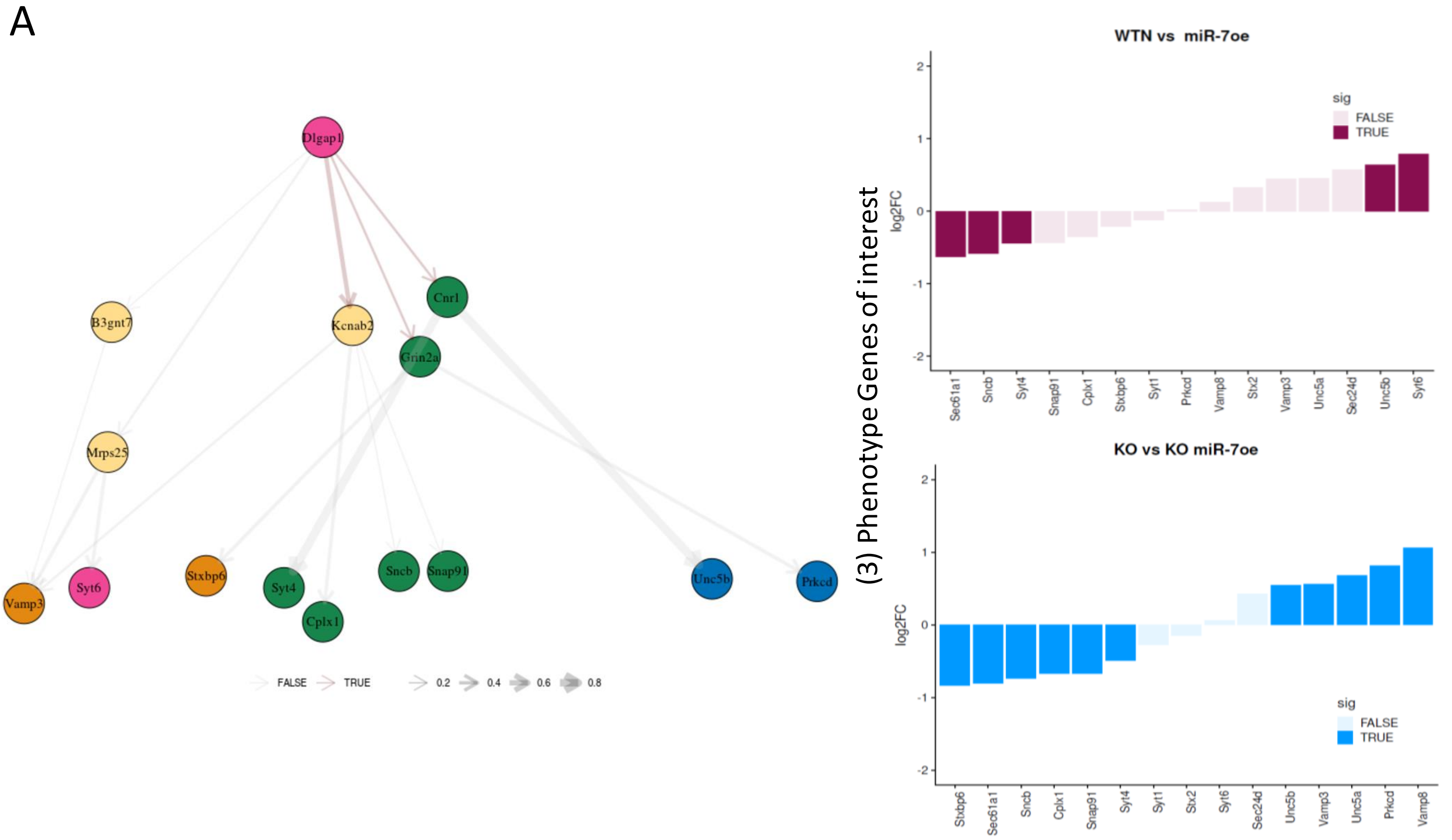

Indirect Regulators

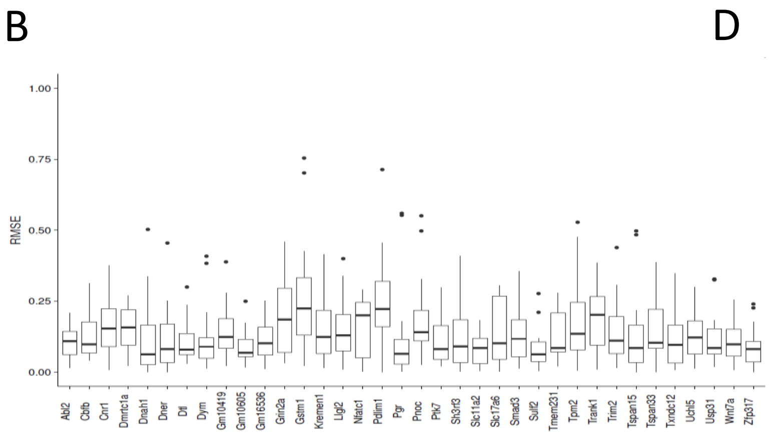

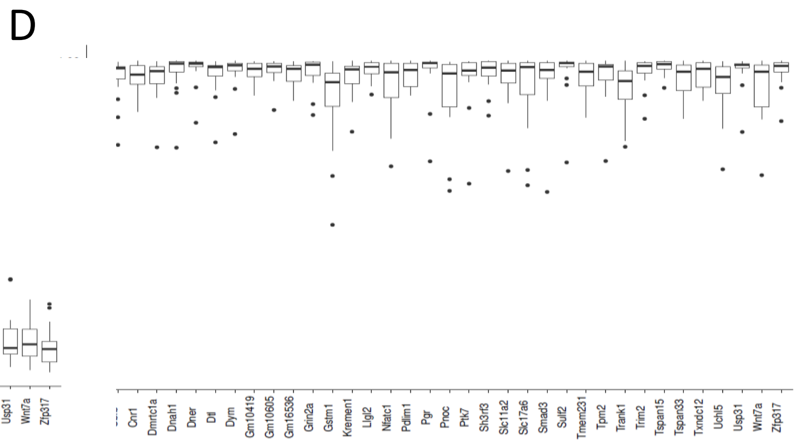

Phenotype Genes of interest

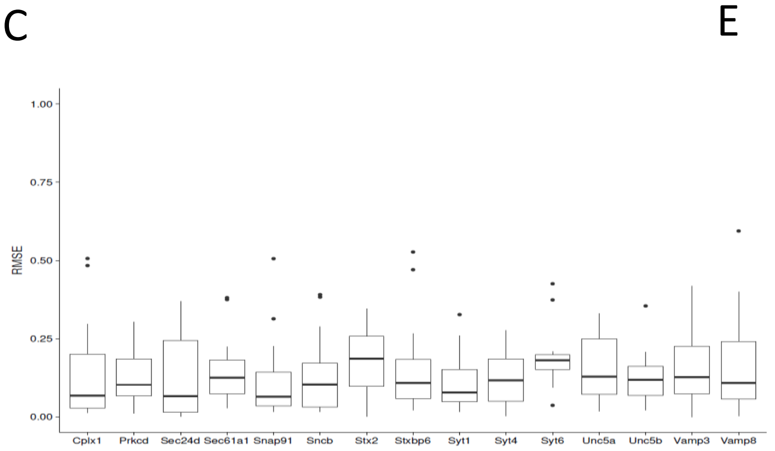

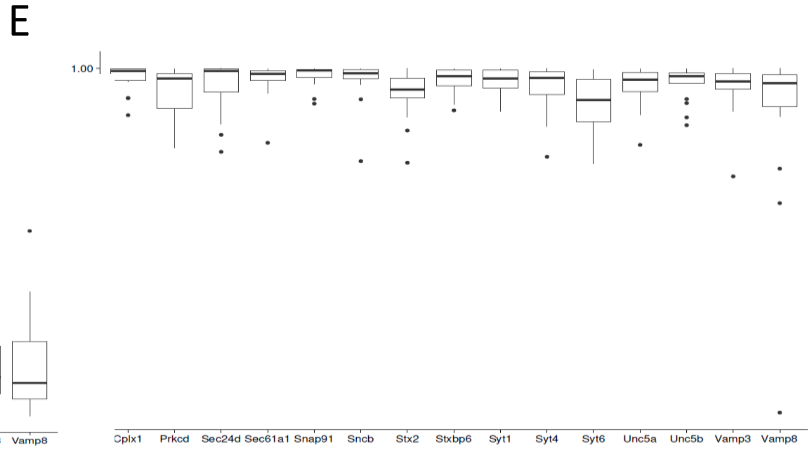

Appendix Figure S7.

(A) Left: Selected connection of the inferred GRN with the largest number of recovered known links from StringDB. Right: Differentially expressed genes in phenotype-related genes of GRN network in each comparison. Bar plots, the mean log2 fold change of 4 independent biological replicates per condition, for each comparison tested. Transparent bars, not significant changes (FDR > 0.05).

(B) Leave-one-out cross-validation performance for "indirect regulators" layer. For each gene, one sample out of 14 was left out and RMSE score was calculated for each of the remaining 13 samples (Methods).

(C) Leave-one-out cross-validation performance for "Phenotype genes of interest" layer. For each gene, RMSE score was calculated as in (D).

(D) Leave-one-out cross-validation performance for "indirect regulators" layer. For each gene, one sample out of 14 was left out and cosine similarity score was calculated for each of the remaining 13 samples (Methods).

(E) Leave-one-out cross-validation performance for "Phenotype genes of interest" layer. For each gene, cosine similarity is calculated as in (F)

Discussion Extended View

Neuronal activity regulated secretion pathways are the key mechanistic tools to control the network connectivity responsible for long-term brain changes (LTP, synaptic morphological changes, neural coding, etc.) and subsequent plastic adaptations (learning, memory, conditioning), where transcriptional and post-transcriptional changes are necessary for the development of neuroplasticity, even though transcriptional target genes that are involved in long-term plasticity have yet to be identified (McClung & Nestler, 2008). Therefore, the importance of this ncRNA regulatory network in an *in vivo* biological context remains to be tested. We believe that finding scenarios where cortical neuronal networks need to adapt quickly and permanently to stressed or highly triggered conditions would be the perfect context to further examine the mechanistic roles of the Cdr1as and miR-7 axis.

Our data show, in primary cortical neurons, miR-7 is expressed at very low levels in resting state (~40 miR-7 molecules per neuron; **Fig. EV4D**), while Cdr1as is very highly expressed (~250 molecules/neuron, **Fig. EV2A**). Nevertheless, we need to be cautions regarding the potential underestimation of molecule counts in smRNA FISH data. This because, even though the imaging ins performed in z-axis stacks, the final dot quantification is done in maximal projection images, which can underrate the real final number of molecules in 3D context. Specifically, for miR-7 we also need to consider that the expression of the mature miRNA is highly control at the transcriptional level, by c-Myc and HoxD10 proteins (Reddy et al., 2008; Chou et al., 2010)); as well as at the processing of the primary transcript, where HuR protein binds to pri-miR-7 conservative terminal parts (Kumar et al., 2017). All of these might result in in the discrepancy between the expression of pri-miR-7 sequence and the expression of mature miR-7 sequence.

According to the description done by Rybak-Wolf et al. (2015) in primary hippocampal neurons Cdr1as expression increases dramatically on DIV14 (120 FC), which could indicate that the role of Cdr1as in forebrain is relevant for neuronal function in mature states of synaptic connections. This positively correlate with our observation of increasing neuronal activity over time in Cdr1as-KO neurons (**Fig. 3)**, where the action of miR-7 must be more strongly buffered.

The precise intracellular pathways activated to achieve Cdr1as up-regulation after stimulation and all observed neuronal responses dependent of miR-7-Cdr1as regulations remain to be uncovered. Some indications of induction of Cdr1as expression by several secretagogues that activate cAMP and PKC signaling pathways (Xu et al., 2015), which in neurons are related to

the activation of metabotropic glutamate receptors (Niciu et al., 2012). In combination with our gene ontology enrichment analysis, which indicate that vesicle targeting and glutamate receptors signaling pathways are affected only in Cdr1as-KO neurons after miR-7 sustained up-regulation (**Fig. 5D**).

A small dysregulation of extremely lowly expressed miR-7, due to the constitutive loss of Cdr1as (**Appendix Fig. S6A-B**) is most likely not reflected in global abundance of mRNAs, because miRNAs are fine-tuners of transcriptome regulations that mostly act under time and stimulus specific circumstances (Bartel, 2004; Schratt, 2009), therefore, only the perturbation of low abundant miR-7 allows us to observe global RNA changes. Unexpected large indirect up-regulation of non-miR-7a-targets genes, can be related with the compensatory mechanism activated by miR-7 to control dysfunctional glutamate secretion of Cdr1as-KO neurons.

Cdr1os (Cdr1as precursor; **Fig. EV5F**), which previously was shown to be up-regulated by the loss of Cdr1as locus in bulk brain tissue (Barrett et al., 2017), is not altered by sustained miR-7 overexpression (**Fig. EV5G, left panel**), an RNA sequencing also confirmed the significant down-regulation of Cyrano in both genotypes caused by miR-7 up-regulation, but not affected by the loss of Cdr1as itself. (**Fig. EV5G, right panel**).

We discovered that sustained miR-7 overexpression in Cdr1as-KO neurons specifically regulates set of genes of functional pathways related to synaptic signaling dynamics, equilibrium of excitatory-inhibitory transmission and synaptic plasticity, and those gene pathway regulations are reflected in modulations of excitatory neurotransmitter release, local and network synaptic activity of the neurons.

Finally, we propose potential gene regulatory pathways for genes related with our functional phenotypes. To identify genes associated with the regulation of neuronal phenotypes of sustained miR-7 expression in neurons with the loss of Cdr1as, we performed Random Forest (RF) analysis (**Appendix Fig. S6A**), which yielded 154 candidate genes across three regulatory layers: (1) miR-7a-5p predicted targets; (2) 'intermediate regulator' genes; (3) phenotype-related genes of interest (**Appendix Fig. S6B**). Then, we used a known protein-protein interaction network (StringDB) to select reliable connections in the predicted gene regulatory network (**red arrows in Appendix Fig. S6B**). Out of the predicted miR-7a-5p target genes, Dlgap1, a postsynaptic scaffold protein in neuronal cells, had the most reliable connections (**Appendix Fig. S7A-E**). *Dlgap1* regulates directly the Kcnab2, Grin2a and Cnr1 genes, which

encode for a potassium channel subunit, a glutamate receptor and a G protein-coupled cannabinoid receptor, respectively. These genes have predicted connection to the following phenotype-related genes: Cplx1, Snap91, Sncb, Vamp3, Prkcd, Stxbp6, which directly induce changes in neurotransmitter secretion. Some of these genes have been previously linked to miR-7-dependent secretion phenotypes (LaPierre et al., 2022; Latreille et al., 2014; Xu et al., 2015).
