# Supplementary figures and images for: miR-7 controls glutamatergic transmission and neuronal connectivity in a Cdr1as-dependent manner
Source: EMBO Rep. 2024 Jun 3;25(7):3008–39. doi: 10.1038/s44319-024-00168-9 (PMC11239925; doi:10.1038/s44319-024-00168-9)

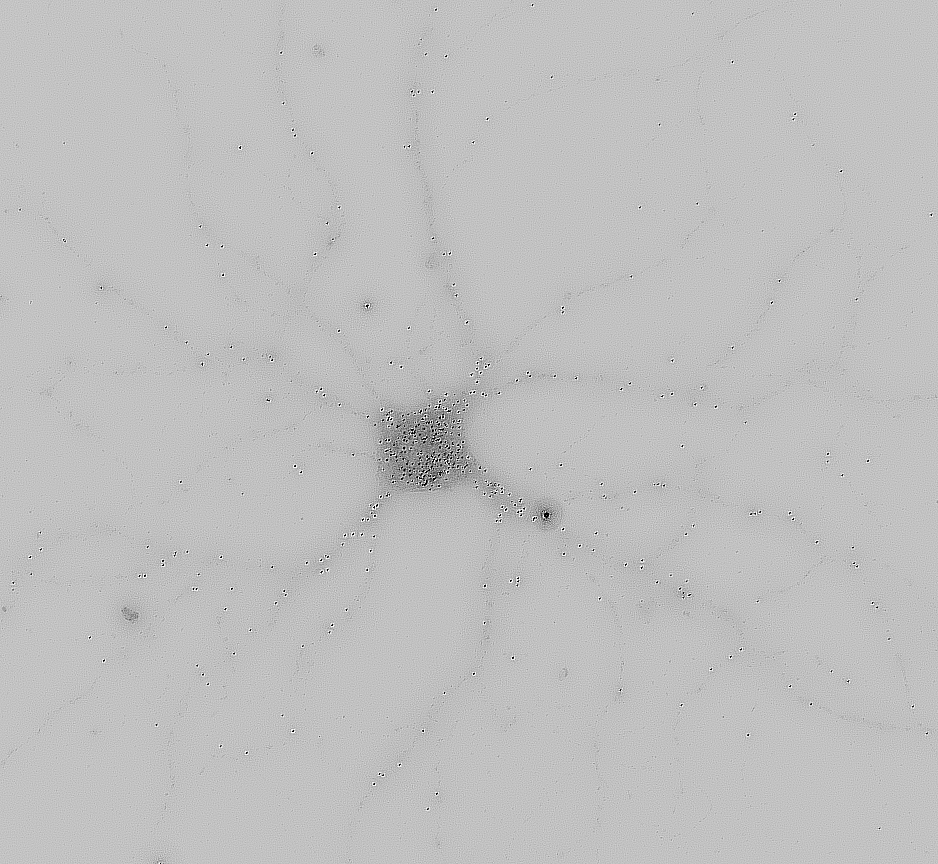

Supplement: Supplementary file 12 — Source data Fig. 4 [file 44319_2024_168_MOESM12_ESM.zip › Cdr1as_control_4A.tif]

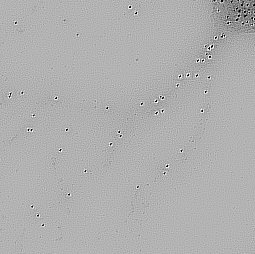

Supplement: Supplementary file 12 — Source data Fig. 4 [file 44319_2024_168_MOESM12_ESM.zip › Cdr1as_control_4A_zoomin.tif]
