# Supplementary material for: miR-7 controls glutamatergic transmission and neuronal connectivity in a Cdr1as-dependent manner
Source: EMBO Rep. 2024 Jun 3;25(7):3008–39. doi: 10.1038/s44319-024-00168-9 (PMC11239925; doi:10.1038/s44319-024-00168-9)
Supplement: Supplementary file 14 — Expanded View Figures [file 44319_2024_168_MOESM14_ESM.pdf]

Expanded View Figures

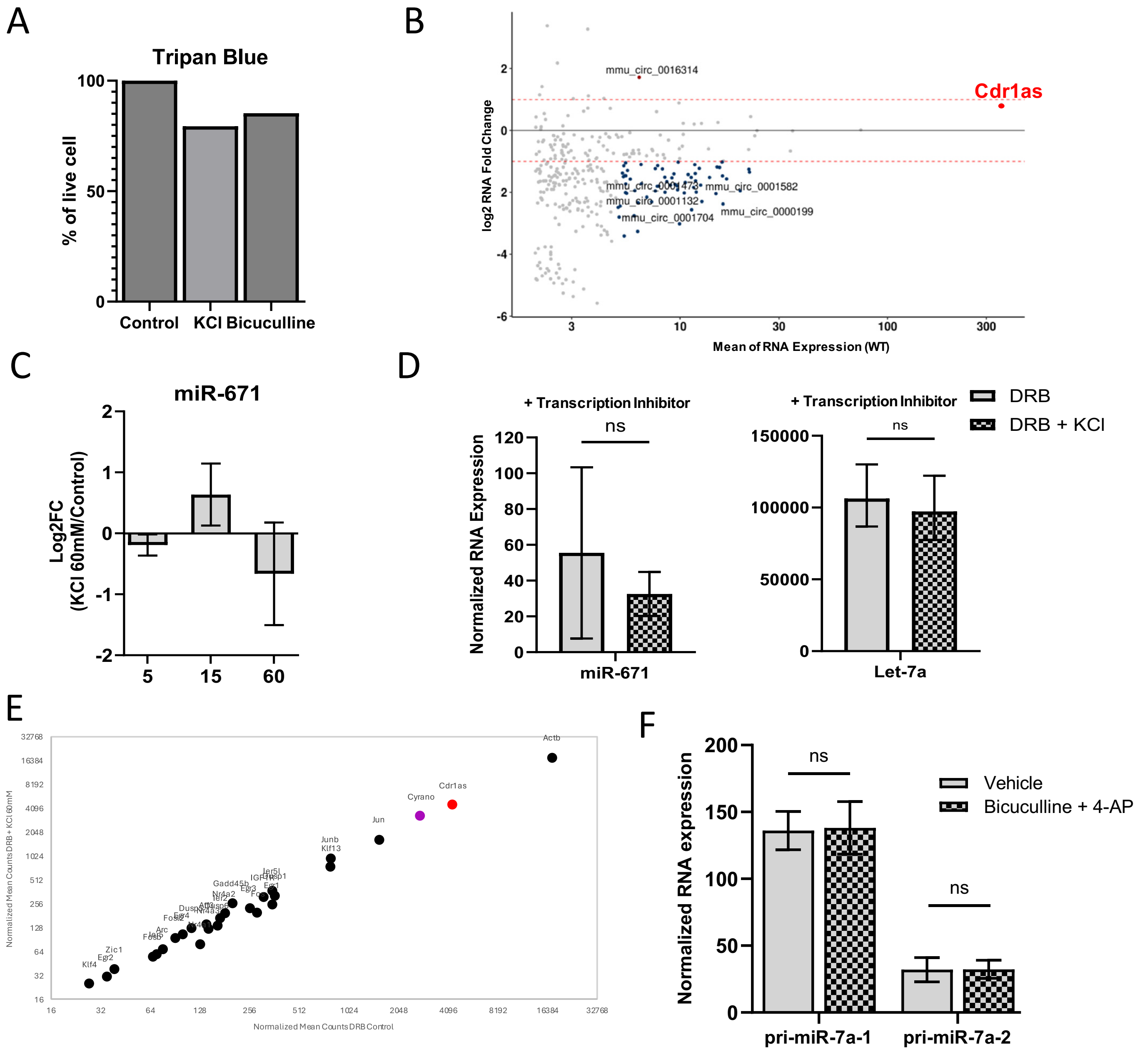

Figure EV1. Sustained neuronal depolarization in neuronal cultures and transcription inhibitor controls.

(**A**) Percentage of live cells after treatments with 60 mM KCl for 1 h (middle bar) or Bicuculine + 4-AP durin 30 min. (right bar), compare with untreated cells as negative control (left bar). Percentage of live cells was stimated based on Tripan blue staining after cell dissociation ("Methods"). (**B**) circRNA expression changes for WT neurons before and after K+ treatment. Plotted is the mean change of 2 independent biological replicates per condition. Red dot: Cdr1as. Blue and brown dots: other statistically significant circRNAs ("Methods"). (**C**) Expression changes of mature miR-671 quantified by TaqMan assay ("Methods"), after 5, 15 and 60 min of sustained KCl depolarization. Bar plot represents the mean of 3 biological replicates (3 independent primary cultures from 3 animals). *P* value: Mann–Whitney U test. Error: SD. (**D**) Expression levels of mature miR-671 (left) and let-7a (right) quantified by small RNA-seq for 3 independent primary cultures, before and after sustained depolarization plus pre-incubation with transcription inhibitor (DRB). *P* value: Mann–Whitney *U* test. Error: SD. (**E**) RNA quantification of IEGs after sustained KCl depolarization plus pre-incubation with transcription inhibitor (DRB) (Nanostring nCounter, "Methods"). RNA counts are normalized to housekeeping genes (Actb, Tubb5 and Vinculin). Cdr1as and Cyrano shown in red and purple, respectively. Each dot represents the mean of 3 biological replicates (3 independent primary cultures from 3 animals). (**F**) Pri-miR-7a-1 (left) and pri-miR-7a-2 (right) RNA expression changes after 30 min incubation with 50 μM Bicuculline + 75 μM 4-Aminopyridine (antagonist of GABA-A receptors and K+ Channel blocker, respectively), compared to corresponding vehicle control (DMSO, gray bars. RNA measured by Nanostring nCounter ("Methods"). RNA counts are normalized to housekeeping genes (Actb, Tubb5 and Vinculin). Bar plot represents the mean of 3 biological replicates (3 independent primary cultures from 3 animals). *P* value: Mann–Whitney *U* test. Error: SD.

 © The Author(s)

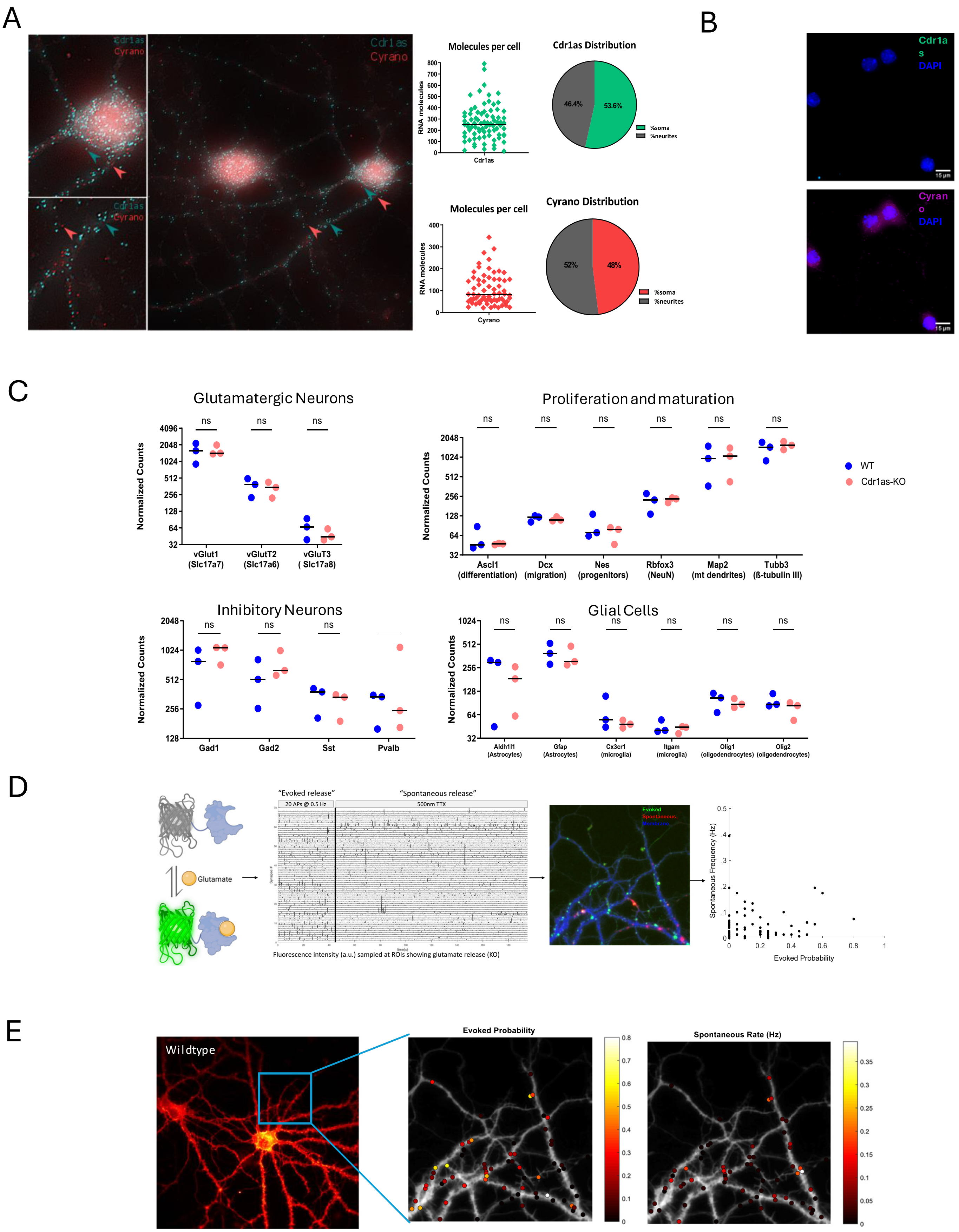
A
Cdr1as
Cyrano
Molecules per cell
RNA molecules
Cdr1as
Cdr1as Distribution
46.4%
53.6%
%soma
%neurites
Molecules per cell
Cyrano
Cyrano Distribution
52%
48%
B
Cdr1as
DAPI
Cyrano
DAPI
C
Glutamatergic Neurons
Normalized Counts
vGlut1 (Slc17a7)
vGlutT2 (Slc17a6)
vGluT3 (Slc17a8)
Proliferation and maturation
Ascl1 (differentiation)
Dcx (migration)
Nes (progenitors)
Rbfox3 (NeuN)
Map2 (mt dendrites)
Tubb3 (ß-tubulin III)
WT
Cdr1as-KO
Inhibitory Neurons
Gad1
Gad2
Sst
Pvalb
Glial Cells
Aldh1l1 (Astrocytes)
Gfap (Astrocytes)
Cx3cr1 (microglia)
Itgam (microglia)
Olig1 (oligodendrocytes)
Olig2 (oligodendrocytes)
ns
D
Glutamate
"Evoked release"
"Spontaneous release"
20 APs @ 0.5 Hz
500nm TTX
Fluorescence intensity (a.u.) sampled at ROIs showing glutamate release (KO)
Evoked
Spontaneous
Spontaneous Frequency (Hz)
Evoked Probability
E
Wildtype
Evoked Probability
Spontaneous Rate (Hz)

◀ **Figure EV2. Characterization of cortical primary neuronal cultures.**

(**A**) Right: Single-molecule RNA FISH (Stellaris, "Methods") of Cdr1as (cyan) and Cyrano (magenta) performed in WT neurons. Left: smRNA FISH quantification of Cdr1as molecules (following Raj et al, 2008, "Methods"). Each dot represents the mean number of molecules in an independent cell (soma + neurites) (Cdr1as $n = 6$; 80 cells) (Cyrano $n = 5$; 69 cells). Horizontal line: Median. Pie charts show molecule distribution in somas versus neurites. (**B**) Control of Cdr1as probe specificity by single-molecule RNA FISH (Stellaris, "Methods") of Cdr1as (cyan) and Cyrano (magenta) Cdr1as-KO neurons DIV21. DAPI: blue. (**C**) Quantification of cellular markers to characterize WT versus Cdr1as-KO primary cultures DIV21 (Nanostring nCounter, "Methods"). RNA counts are normalized to housekeeping genes (Actb, Tubb5 and Vinculin). Each dot represents an independent biological replicate (3 independent primary cultures from 3 animals). Excitatory neurons (Sclc17a7, Sclc17a6, Sclc17a8), Inhibitory neurons (*Gad1, Gad2, Sst, Pvalb*), Proliferation and maturation markers (*Ascl1, Dxc, Nes, Rbfox3, Map2, Tubb3*) and Glial cells markers (*Aldh1, Gfap, Cx3cr1, Itgam, Olig1, Olig2*) are plotted. *P* value: Mann–Whitney U test. Horizontal bar: Median. (**D**) Transduction of a glutamate sensor (AAV, "Methods") into WT and Cdr1as-KO primary neurons followed by real-time imaging of excitatory synaptic terminals during AP-evoked (20 APs at 0.5 Hz) and spontaneous (5 min + 500 nM TTX) release conditions. (**E**) Visualization of glutamate sensor expression (GlusnFR) in WT primary neuron DIV20 (representative image). Region of interest zoom-in (yellow box). Middle and right panels indicate selection of active synaptic terminals and quantification of evoked probability and spontaneous frequency, respectively.

 © The Author(s)

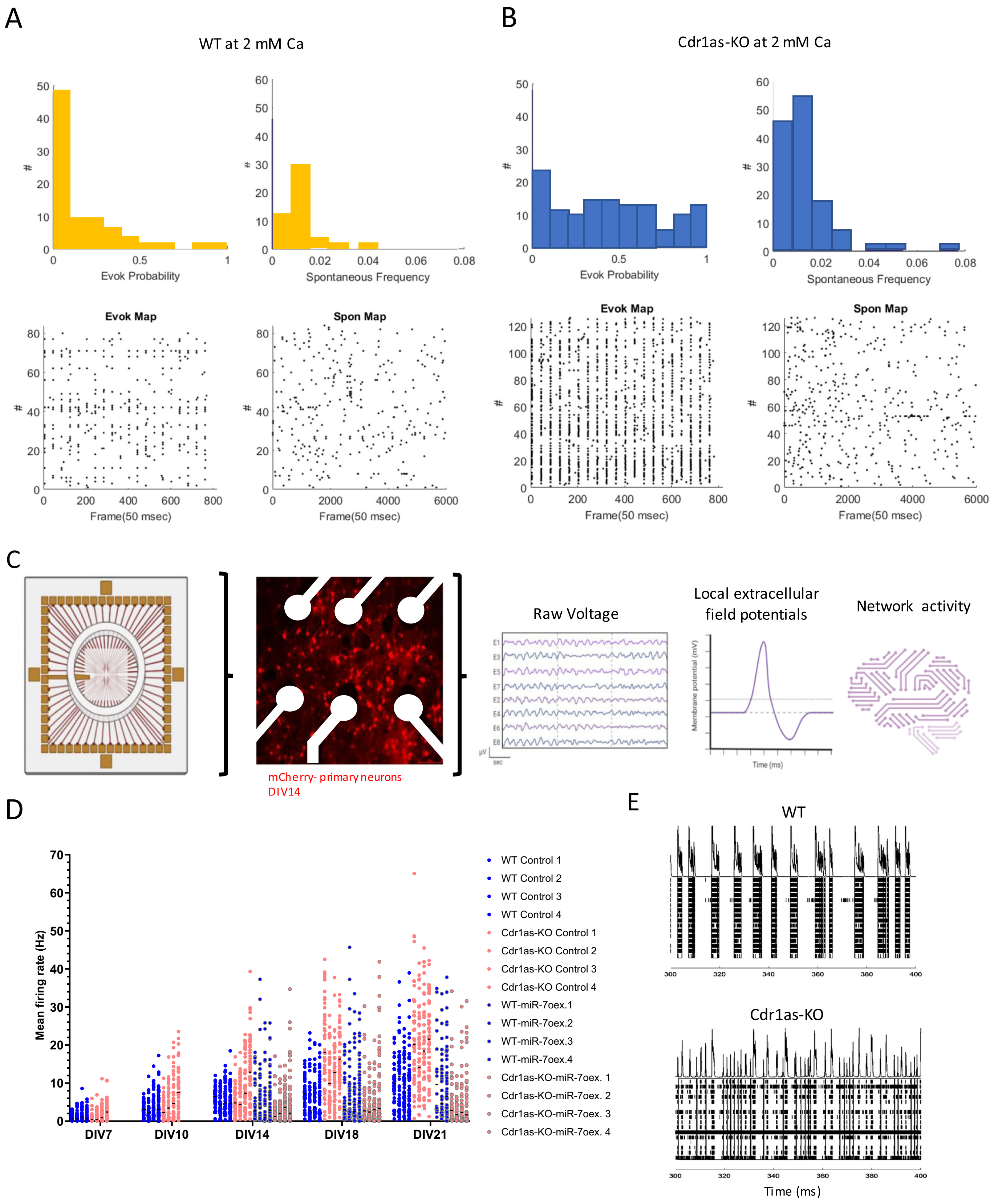
A
WT at 2 mM Ca
Evok Probability
Spontaneous Frequency
Evok Map
Spon Map
Frame(50 msec)
B
Cdr1as-KO at 2 mM Ca
Evok Probability
Spontaneous Frequency
Evok Map
Spon Map
Frame(50 msec)
C
mCherry- primary neurons
DIV14
Raw Voltage
Local extracellular
field potentials
Network activity
Membrane potential (mV)
Time (ms)
D
Mean firing rate (Hz)
DIV7
DIV10
DIV14
DIV18
DIV21
WT Control 1
WT Control 2
WT Control 3
WT Control 4
Cdr1as-KO Control 1
Cdr1as-KO Control 2
Cdr1as-KO Control 3
Cdr1as-KO Control 4
WT-miR-7oex.1
WT-miR-7oex.2
WT-miR-7oex.3
WT-miR-7oex.4
Cdr1as-KO-miR-7oex. 1
Cdr1as-KO-miR-7oex. 2
Cdr1as-KO-miR-7oex. 3
Cdr1as-KO-miR-7oex. 4
E
WT
Cdr1as-KO
Time (ms)

◀ **Figure EV3. Glutamate release and multielectrode array (MEA) method and quality controls.**

(**A**) Histogram and scatter plot show distribution of number of active synapses of WT neurons cultured in media with 2 mM Ca^{+2} to ensure neuronal firing. AP-evoked glutamate release calculated as evoked probability (left) and spontaneous glutamate release calculated as spontaneous frequency (right), respectively. (**B**) Histogram and scatter plot show distribution of number of active synapses of Cdr1as-KO neurons cultured in media with 2 mM Ca^{+2} to ensure neuronal firing. AP-evoked glutamate release calculated as evoked probability (left) and spontaneous glutamate release calculated as spontaneous frequency (right), respectively. (**C**) Scheme of the Multi-electrode Array recording protocol (Axion Biosystems, CytoView MEA 48, "Methods"). Second panel: representative image of cultured neurons DIV14 in a recording well. white: electrodes, red: mCherry reporter. Third panel: schematic representation of output data, extracellular field potentials and neuronal network activity. AP: Adaptative threshold 6 SD. Sampling frequency 12.5 kHz. Active electrode selection criteria 5 spikes/minute. (**D**) Mean Firing Rate: Total number of spikes per single electrode divided by the duration of the analysis (600 s), in Hz. Each column represents recordings from a single biological replicate. Each dot represents recordings from a single electrode. (**E**) Example raw spikes from multi electrodes Array recording: 100 ms raw spikes of WT and Cdr1as-KO neurons DIV21. Each row represents one independent electrode (black bars).

 © The Author(s)

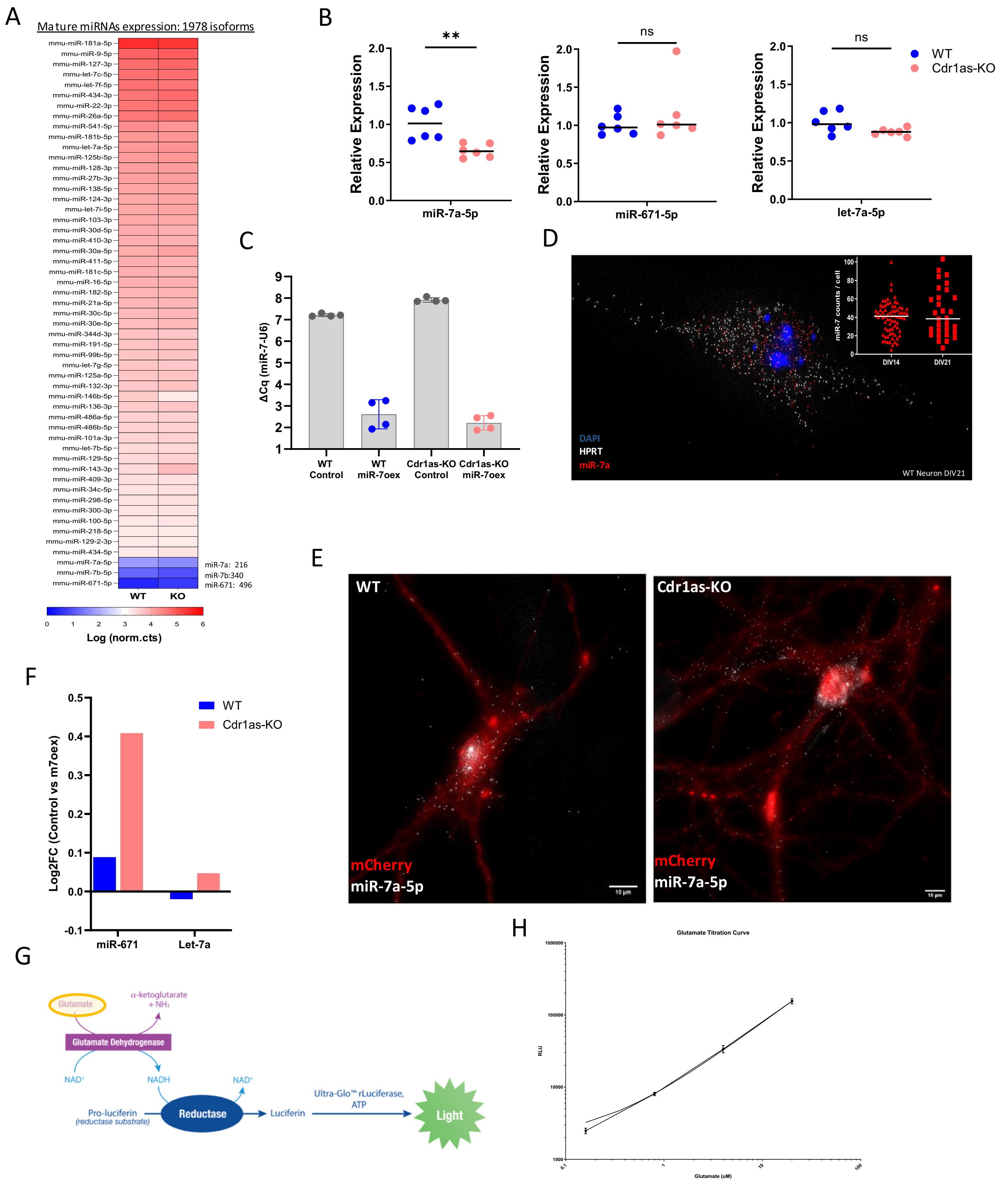
A
Mature miRNAs expression: 1978 isoforms
mmu-miR-181a-5p
mmu-miR-9-5p
mmu-miR-127-3p
mmu-let-7c-5p
mmu-let-7f-5p
mmu-miR-434-3p
mmu-miR-22-3p
mmu-miR-26a-5p
mmu-miR-541-5p
mmu-miR-181b-5p
mmu-let-7a-5p
mmu-miR-125b-5p
mmu-miR-128-3p
mmu-miR-27b-3p
mmu-miR-138-5p
mmu-miR-124-3p
mmu-let-7i-5p
mmu-miR-103-3p
mmu-miR-30d-5p
mmu-miR-410-3p
mmu-miR-30a-5p
mmu-miR-411-5p
mmu-miR-181c-5p
mmu-miR-16-5p
mmu-miR-182-5p
mmu-miR-21a-5p
mmu-miR-30c-5p
mmu-miR-30e-5p
mmu-miR-344d-3p
mmu-miR-191-5p
mmu-miR-99b-5p
mmu-let-7g-5p
mmu-miR-125a-5p
mmu-miR-132-3p
mmu-miR-146b-5p
mmu-miR-136-3p
mmu-miR-486a-5p
mmu-miR-486b-5p
mmu-miR-101a-3p
mmu-let-7b-5p
mmu-miR-129-5p
mmu-miR-143-3p
mmu-miR-409-3p
mmu-miR-34c-5p
mmu-miR-298-5p
mmu-miR-300-3p
mmu-miR-100-5p
mmu-miR-218-5p
mmu-miR-129-2-3p
mmu-miR-434-5p
mmu-miR-7a-5p
mmu-miR-7b-5p
mmu-miR-671-5p
miR-7a: 216
miR-7b:340
miR-671: 496
WT
KO
0 1 2 3 4 5 6
Log (norm.cts)
B
Relative Expression
**
miR-7a-5p
ns
miR-671-5p
ns
let-7a-5p
WT
Cdr1as-KO
C
ΔCq (miR-7-U6)
WT Control
WT miR-7oex
Cdr1as-KO Control
Cdr1as-KO miR-7oex
D
miR-7 counts / cell
DIV14
DIV21
DAPI
HPRT
miR-7a
WT Neuron DIV21
E
WT
Cdr1as-KO
mCherry
miR-7a-5p
10 µm
F
Log2FC (Control vs m7oex)
WT
Cdr1as-KO
miR-671
Let-7a
G
Glutamate
α-ketoglutarate + NH3
Glutamate Dehydrogenase
NAD+
NADH
NAD+
Pro-luciferin (reductase substrate)
Reductase
Luciferin
Ultra-Glo™ rLuciferase, ATP
Light
H
Glutamate Titration Curve
RLU
Glutamate (uM)

◀ **Figure EV4. miRNAs in WT and Cdr1as-KO cortical neurons.**

(**A**) Heatmap from bulk smRNA-Seq ("Methods") (1978 miRNA isoforms detected), normalized expression plotted (log.norm.counts) of top 50 mature miRNAs in WT and Cdr1as-KO primary neurons DIV21, plus miR-7a, miR-7b and miR-671. (**B**) Quantification of mature miR-7a-5p, mir-671-5p and let-7a-5p (TaqMan Assay, "Methods") to characterize WT versus Cdr1as-KO primary cultures DIV21. RNA normalized to housekeeping genes (snRNA U6, snoRNA202) Each dot represents an independent biological replicate (6 independent primary cultures from 6 animals). *P* value: Mann–Whitney *U* test. Horizontal line: Median. (**C**) Quantification of mature miR-7a-5p (TaqMan Assay, "Methods") in WT and Cdr1as-KO primary cultures at 14 days post miR-7 overexpression. RNA normalized to housekeeping gene snRNA U6. Each dot represents an independent biological replicate (4 independent primary cultures from 4 animals). (**D**) Single-molecule RNA FISH (ViewRNA Plus, "Methods") of miR-7a-5p (red) and housekeeping gene Hprt (white) performed in WT neurons DIV14 and DIV21, DAPI: blue. Insert: smRNA FISH quantification of miR-7 molecules (following Raj et al, 2008, "Methods"). Each dot represents the mean number of molecules in an independent cell: DIV14 ($n = 75$ cells) and DIV21 ($n = 30$ cells). (Widefield microscopy 60×). (**E**) Single-molecule miRNA in situ hybridization (ViewRNA Plus, "Methods") of miR-7a-5p (white) and infection reporter mCherry (red), performed in WT and Cdr1as-KO neurons DIV21, 14 days post miR-7 overexpression (Widefield microscopy 60×). (**F**) Quantification of miR-671 and let-7a after miR-7 overexpression (14dpi) by RNA-Seq ("Methods") in WT and Cdr1as-KO neurons at DIV21. Bar plots represents mean of 4 independent biological replicates per genotype. (**G**) Schematic representation of the enzymatic principle behind glutamate secretion assay. Modified from Glutamate-Glo™ Assay ("Methods"). GDH enzyme catalyzes the oxidation of glutamate with associated reduction of NAD+ to NADH. In the presence of NADH, Reductase enzymatically reduces a pro-luciferin to luciferin. Luciferin using Ultra-Glo™ Luciferase and ATP, and the amount of light produced (RLU) is proportional to the amount of glutamate in the sample. (**H**) Standard curve for calibration of glutamate secretion assay. Serial dilutions curve of 50 µM Glutamate stock solution. Quantification of secreted glutamate concentrations for tested samples based on interpolation of RLU values.

 © The Author(s)

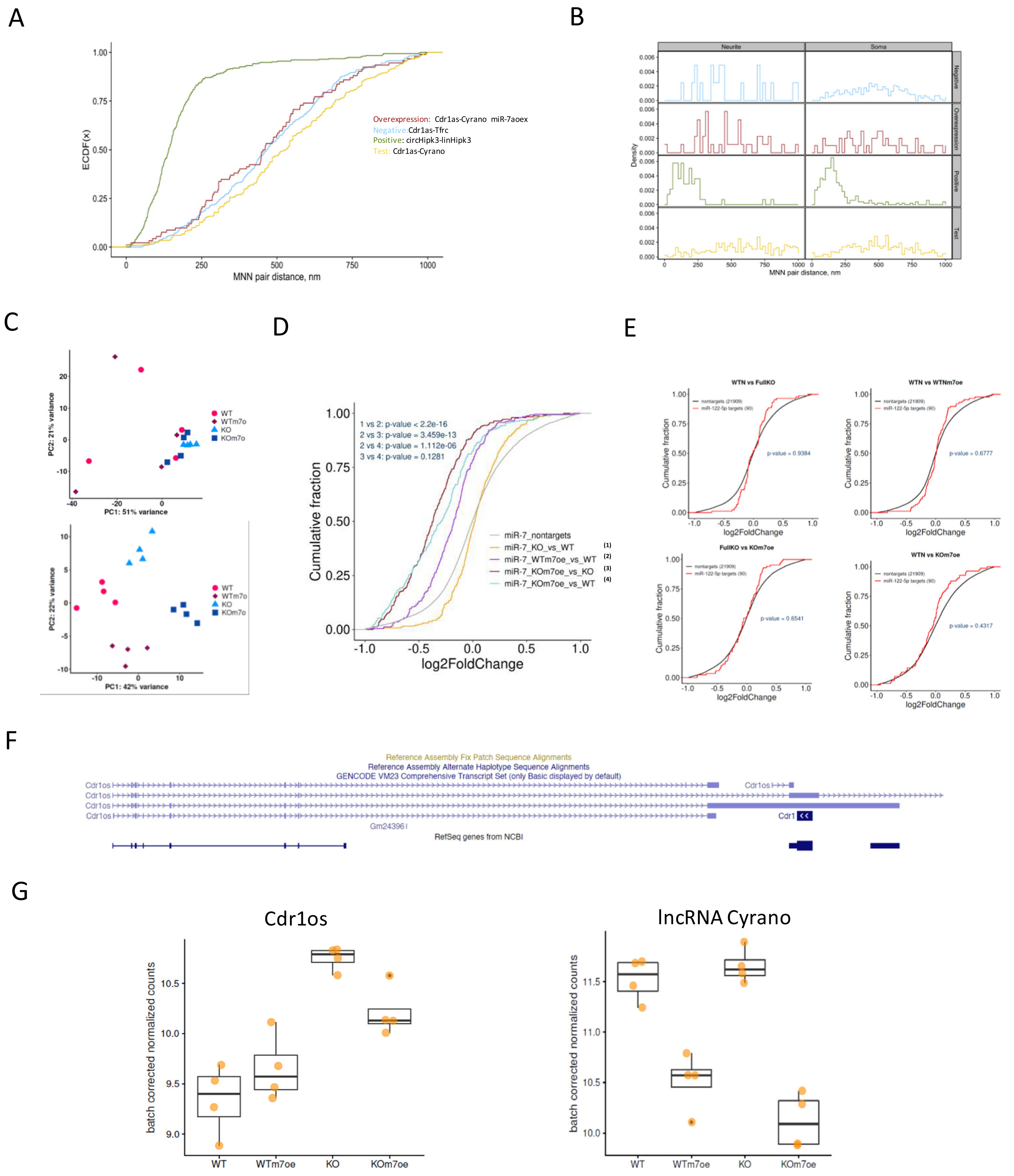
A
B
C
D
E
F
G
Overexpression: Cdr1as-Cyrano miR-7aoex
Negative: Cdr1as-Tfrc
Positive: circHipk3-linHipk3
Test: Cdr1as-Cyrano
ECDF(x)
MNN pair distance, nm
Neurite
Soma
Negative
Overexpression
Positive
Test
Density
PC1: 51% variance
PC2: 21% variance
PC1: 42% variance
PC2: 22% variance
WT
WTm7o
KO
KOm7o
1 vs 2: p-value < 2.2e-16
2 vs 3: p-value = 3.459e-13
2 vs 4: p-value = 1.112e-06
3 vs 4: p-value = 0.1281
Cumulative fraction
log2FoldChange
miR-7_nontargets
miR-7_KO_vs_WT (1)
miR-7_WTm7oe_vs_WT (2)
miR-7_KOm7oe_vs_KO (3)
miR-7_KOm7oe_vs_WT (4)
WTN vs FullKO
WTN vs WTNm7oe
FullKO vs KOm7oe
WTN vs KOm7oe
p-value = 0.9384
p-value = 0.6777
p-value = 0.6541
p-value = 0.4317
Reference Assembly Fix Patch Sequence Alignments
Reference Assembly Alternate Haplotype Sequence Alignments
GENCODE VM23 Comprehensive Transcript Set (only Basic displayed by default)
Cdr1os
Cdr1
Gm24396
RefSeq genes from NCBI
Cdr1os
lncRNA Cyrano
batch corrected normalized counts
WT
WTm7oe
KO
KOm7oe

Figure EV5. Cdr1as and Cyrano colocalization analysis. miR-7 everexpression mRNA-sequencing quality controls.

(**A**) Cumulative distribution function (CDF) plot of molecule distances based on smRNA FISH images of WT neurons DIV21. before and after miR-7 overexpression ("Methods") comparing all computationally predicted mutual nearest neighbor distances in nm all conditions (MNN, "Methods"). Positive technical control: circHipk3-linHipk3; Negative control: Cdr1as-Tfrc; Test: Cdr1as-Cyrano; overexpression: Cdr1as-Cyrano miR-7aoex. 2 independent biological replicates from 2 animals. (**B**) Density plot molecule distances based on smRNA FISH to compare somas versus neurites for all tested conditions. Analysis conditions same as in (**A**). (**C**) Principal component analysis (PCA) of all data sets. Each replicate represented by one dot. Original (up) and batch-corrected with nested design (down). (**D**) Cumulative distribution function (CDF) plot of gene expression comparing all computationally predicted miR-7 targets ("Methods") to mRNAs lacking predicted miR-7 target sites (non-targets, gray), across 4 independent biological replicates of WT, Cdr1as-KO, WT + miR-7 overexpression, Cdr1as-KO + miR-7 overexpression. *P* value: *U* Mann–Whitney test. (**E**) Cumulative distribution function (CDF) plot of gene expression comparing all computationally predicted miR-122 targets ("Methods", red) to mRNAs lacking predicted miR-7 target sites (non-targets, black), across 4 independent biological replicates of WT, Cdr1as-KO, WT + miR-7 overexpression, Cdr1as-KO + miR-7 overexpression. *P* value: *U* Mann–Whitney test. (**F**) Cdr1os transcript (Cdr1as precursor transcriptional unit) reference sequence alignments from Genome Browser (GENCODE VM23). (**G**) Gene expression of Cdr1os and lncRNA Cyrano in each dataset. Box plots, 4 independent biological replicates per condition, for each comparison tested. (FDR < 0.05).

 © The Author(s)
